# Patterns of Emergency Care for Possible Acute Coronary Syndrome Among Patients with Chest Pain or Shortness of Breath at a Tanzanian Referral Hospital

**DOI:** 10.5334/gh.402

**Published:** 2020-02-06

**Authors:** Julian T. Hertz, Godfrey L. Kweka, Gerald S. Bloomfield, Alexander T. Limkakeng, Zak Loring, Gloria Temu, Blandina T. Mmbaga, Charles J. Gerardo, Francis M. Sakita

**Affiliations:** 1 Division of Emergency Medicine, Duke University School of Medicine, Durham, NC, US; 2 Duke Global Health Institute, US; 3 Kilimanjaro Clinical Research Institute, Moshi, TZ; 4 Division of Cardiology, Duke University School of Medicine, NC, US; 5 Duke Clinical Research Institute, NC, US; 6 Department of Medicine, Kilimanjaro Christian Medical Centre, Moshi, TZ; 7 Kilimanjaro Christian Medical University College, Moshi, TZ; 8 Department of Emergency Medicine, Kilimanjaro Christian Medical Centre, Moshi, TZ

**Keywords:** acute coronary syndrome, sub-Saharan Africa, Tanzania, emergency department

## Abstract

**Background:**

Acute coronary syndrome (ACS) is thought to be a rare diagnosis in sub-Saharan Africa, but little is known about diagnostic practices for patients with possible ACS symptoms in the region.

**Objective:**

To describe current care practices for patients with ACS symptoms in Tanzania to identify factors that may contribute to ACS under-detection.

**Methods:**

Emergency department patients with chest pain or shortness of breath at a Tanzanian referral hospital were prospectively observed. Medical histories were obtained, and diagnostic workups, treatments, and diagnoses were recorded. Five-year risk of cardiovascular events was calculated via the Harvard National Health and Nutrition Examination Survey risk score. Telephone follow-ups were conducted 30 days after enrollment.

**Results:**

Of 339 enrolled patients, the median (IQR) age was 60 (46, 72) years, 252 (74.3%) had hypertension, and 222 (65.5%) had >10% five-year risk of cardiovascular event. The median duration of symptoms prior to presentation was 7 days, and 314 (92.6%) reported symptoms worsened by exertion. Of participants, 170 (50.1%) received an electrocardiogram, and 9 (2.7%) underwent cardiac biomarker testing. There was no univariate association between five-year cardiovascular risk and decision to obtain an electrocardiogram (*p* = 0.595). The most common physician-documented diagnoses were symptomatic hypertension (104 patients, 30.7%) and heart failure (99 patients, 29.2%). Six patients (1.8%) were diagnosed with ACS, and 3 (0.9%) received aspirin. Among 284 (83.8%) patients completing 30-day follow-up, 20 (7.0%) had died.

**Conclusions:**

Many patients with ACS risk factors present to the emergency department of a Tanzanian referral hospital with possible ACS symptoms, but marked delays in care-seeking are common. Complete diagnostic workups for ACS are uncommon, ACS is rarely diagnosed or treated with evidence-based therapies, and mortality in patients with these symptoms is high. Physician practices may be contributing to ACS under-detection in Tanzania, and interventions are needed to improve ACS care.

## Introduction

Acute coronary syndrome (ACS) is a life-threatening emergency and a leading cause of death and disability worldwide [24]. In sub-Saharan Africa (SSA), the incidence of ACS is presumed to be increasing amid the rising prevalence of risk factors such as hypertension and obesity [12]. In Tanzania, for example, ACS is currently estimated to be the fourth leading cause of death in the country [18]. Despite such estimates, ACS remains a rare diagnosis across SSA [2,13], a fact that has perplexed epidemiologists [17,26]. Some have speculated that the disease may be under-diagnosed due to a combination of limited community awareness of ACS, inadequate clinician training, and lack of diagnostic equipment [4,14,26].

However, little is known about patterns of ACS care in SSA. In settings outside SSA, ACS symptoms such as chest pain or shortness of breath are common among emergency department (ED) patients, and diagnostic workups for ACS are routine [5,8,21]. In contrast, in a single-center study in urban Tanzania, chest pain was present in only 1.3% of adult ED patients and 9.6% of those with chest pain were diagnosed with ACS [23]. However, beyond this single study, there is currently limited data regarding the prevalence of chest pain among ED patients, the risk profiles of such patients, and the diagnostic workups and treatments given to such patients in SSA. Describing such patterns of care is essential to understanding the role of physician behaviors in ACS under-detection in SSA.

The purpose of this study was to determine the prevalence of chest pain and shortness of breath among adults presenting to the ED in northern Tanzania, to characterize the cardiovascular risk profile of this population, and to describe patterns of diagnostic workups and treatments for ACS among these patients.

## Methods

### Study Setting

This study was conducted in the ED at Kilimanjaro Christian Medical Center (KCMC), a tertiary care center in Moshi, Tanzania. In 2014, the local prevalence of hypertension and diabetes was estimated to be 28% and 6%, respectively [10,29]. KCMC has multiple electrocardiogram (ECG) machines, and the laboratory can perform laboratory-based assays for troponin I and troponin T. KCMC has capacity for thrombolytic therapy, but does not presently have capacity for cardiac catheterization or stress testing.

### Study Procedures

A prospective observational study was conducted from 20 August 2018 through 4 January 2019. All adult ED patients (age ≥ 18 years) were screened by trained research assistants. Screening was conducted during one shift (morning, evening, or overnight) per day, with the number of days assigned to each shift proportional to total patient volumes during those shifts. Patients with primary or secondary complaint of chest pain or shortness of breath were considered for inclusion. Exclusion criteria were self-reported fever, chest pain secondary to physical trauma, and inability to provide informed consent. A standardized questionnaire regarding sociodemographic features and medical history derived from the World Health Organization (WHO) STEPS instrument was administered to all participants [31]. Weight and height were measured for each participant and blood pressure was measured using the Beurer BM40 automatic blood pressure monitor (Beurer, Ulm, Germany). Patients were followed from time of enrollment to time of ED disposition (admission or discharge), and all diagnostic investigations or treatments ordered were recorded. Patient diagnoses were recorded directly from the written patient chart, and any prescriptions written by the ED physician were also recorded. Thirty days after enrollment, participants were contacted via telephone to assess vital status and current state of symptoms. If patients were deceased or unavailable, the follow-up survey was administered to a relative. All study instruments were forward- and back-translated from English to Swahili in order to ensure content fidelity and clarity.

### Study Definitions

Hypertension was defined as a self-reported history of hypertension or a measured blood pressure of ≥ 140/90 mmHg at time of enrollment, as per the JNC 8 panel recommendations [19]. History of diabetes, hyperlipidemia, HIV infection, history of tobacco use, and personal or family history of cardiovascular disease were obtained by patient self-report. Obesity was defined as body mass index (BMI) ≥ 30 kg/m^2^, overweight as 30 kg/m^2^ > BMI ≥ 25 kg/m^2^, normal weight as 25 kg/m^2^ > BMI ≥ 17.5 kg/m^2^, and underweight as BMI < 17.5 kg/m^2^. Poor diet was defined as not eating vegetables and fruits at least once daily, based on the published association between daily consumption of vegetables and fruits and reduced risk of cardiovascular events [3]. Sedentary lifestyle was defined as self-report of less than 150 minutes of moderately vigorous physical activity per week, as per WHO guidelines [30]. Self-reported severity of angina was defined according to Canadian Cardiovascular Society (CCS) guidelines for the grading of angina pectoris [7]. Patients with documented diagnosis of ACS, myocardial infarction, or unstable angina were defined as cases of ACS. Patients with documented diagnosis of hypertensive emergency, hypertensive urgency, or severe hypertension were defined as cases of symptomatic hypertension. Duration of hospitalization was defined by patient self-report on 30-day follow-up.

### Statistical Analyses

All statistical analyses were performed in RStudio (RStudio, Boston, MA, USA). BMI was calculated directly from measured patient weight and height. Five-year risk of cardiovascular event was determined for each patient using the internationally-validated Harvard National Health and Nutrition Examination Survey risk score based on age, systolic blood pressure, sex, current smoking, diabetes, and BMI [11]. Associations between categorical variables were analyzed with Pearson’s chi-squared, and associations between continuous and categorical variables were assessed using the Welch t-test. Univariate odds ratios and corresponding 95% confidence intervals were calculated from contingency tables to assess the magnitude of associations between categorical variables. Multivariate logistic regression was performed to identify predictors of physician decision to obtain an ECG. The pool of potential predictor variables for multivariate regression was determined *a priori* based on plausibility of a causal association with decision to obtain an ECG. Any predictor variable with evidence of univariate association with decision to obtain an ECG (*p* < 0.10) was retained in the multivariate model; age, sex, hypertension, and diabetes were also forced into the regression model regardless of *p*-value. All analyses were conducted at a significance threshold of 5%.

### Ethics Statement

The study protocol conformed to the ethical guidelines of the 1975 Declaration of Helsinki, and this study received ethics approval from the Duke Health Institutional Review Board, the Kilimanjaro Christian Medical Centre Research Ethics Committee, and the Tanzania National Institutes for Medical Research Ethics Coordinating Committee. All participants provided written informed consent prior to enrollment.

## Results

**Figure 1:**
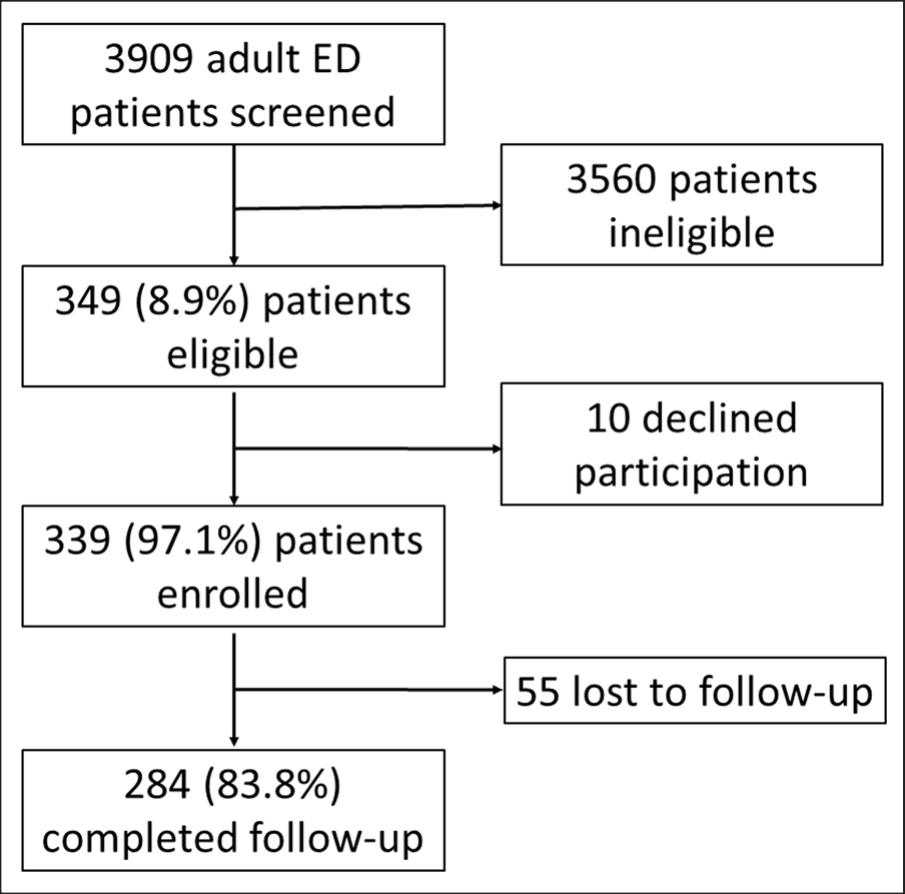
Flow diagram of study participants.

During the study period, 3,909 adult ED patients were screened, of whom 349 (8.9%) were eligible for inclusion, with 339 (97.1%) consenting to study enrollment (Figure 1). The median (IQR) age of enrolled patients was 60 (46, 72) years and 144 (42.5%) were male (Table 1). Of participants, 252 (74.3%) met the study definition for hypertension, 156 (46.0%) were overweight or obese, and 304 (89.7%) met the study definition for poor diet. Regarding overall cardiovascular risk, 222 (65.5%) patients had greater than 10% five-year risk of cardiovascular event.

**Table 1:** Characteristics and cardiovascular risk factors among emergency department patients with chest pain or shortness of breath, northern Tanzania, 2018 (N = 339).

Patient Characteristics	Median	(IQR)

Age, years	60	(46, 72)
Systolic blood pressure, mmHg	138	(121, 155)
Diastolic blood pressure, mmHg	85	(72, 96)
	**Number of patients**	**(%)**

Male	144	(42.5)
Hypertension	252	(74.3)
Diabetes	44	(13.0)
Hyperlipidemia	39	(11.5)
History of tobacco use	109	(32.2)
Body mass index		
Underweight	28	(8.3)
Normal weight	155	(45.7)
Overweight	85	(25.1)
Obese	71	(20.9)
Self-reported history of CVD	18	(5.3)
Family history of CVD	86	(25.4)
Poor diet	304	(89.7)
Sedentary lifestyle	124	(36.6)
Education		
Primary or none	241	(71.1)
Secondary or university	98	(28.9)
HIV infected	6	(1.8)
Taking aspirin daily	34	(10.0)
5-year risk of cardiovascular event		
<5%	74	(21.8)
5–10%	43	(12.7)
10–20%	76	(22.4)
20–30%	75	(22.1)
>30%	71	(20.9)

CVD: Cardiovascular disease. HIV: Human immunodeficiency virus.

The most common primary complaints among patients were chest pain and shortness of breath, endorsed by 122 (36.0%) and 107 (31.5%) participants, respectively (Table 2). Of all ED patients screened, 261 (6.7%) had chest pain and 244 (6.2%) had shortness of breath. The median (IQR) duration of illness prior to hospital presentation was 7 (3, 28) days, and 314 (92.6%) reported symptoms worse with exertion.

**Table 2:** Features of present illness among emergency department patients with chest pain or shortness of breath, northern Tanzania, 2018 (N = 339).

	Number of patients	(%)

Primary complaint*		
Chest pain	122	(36.0)
Shortness of breath	107	(31.5)
Palpitations	18	(5.3)
Leg swelling	11	(3.2)
Abdominal pain	10	(2.9)
Other	71	(20.9)
Secondary complaints*		
Chest pain	139	(41.0)
Shortness of breath	137	(40.4)
Abdominal pain	66	(19.5)
Palpitations	60	(17.7)
Cough	60	(17.7)
Back pain	56	(16.5)
Leg swelling	51	(15.0)
Headache	38	(11.2)
Arm/jaw pain	35	(10.3)
Generalized weakness	28	(8.3)
Other	110	(32.4)
Symptom duration prior to presentation, median (IQR), days	7	(3, 28)
CCS grading for anginal severity		
Non-exertional symptoms	25	(7.4)
Class I	38	(11.2)
Class II	95	(28.0)
Class III	74	(21.8)
Class IV	107	(31.6)

* Only one primary complaint was allowed per patient, but patients could name multiple secondary complaints. CCS: Canadian Cardiovascular Society [20].

**Figure 2:**
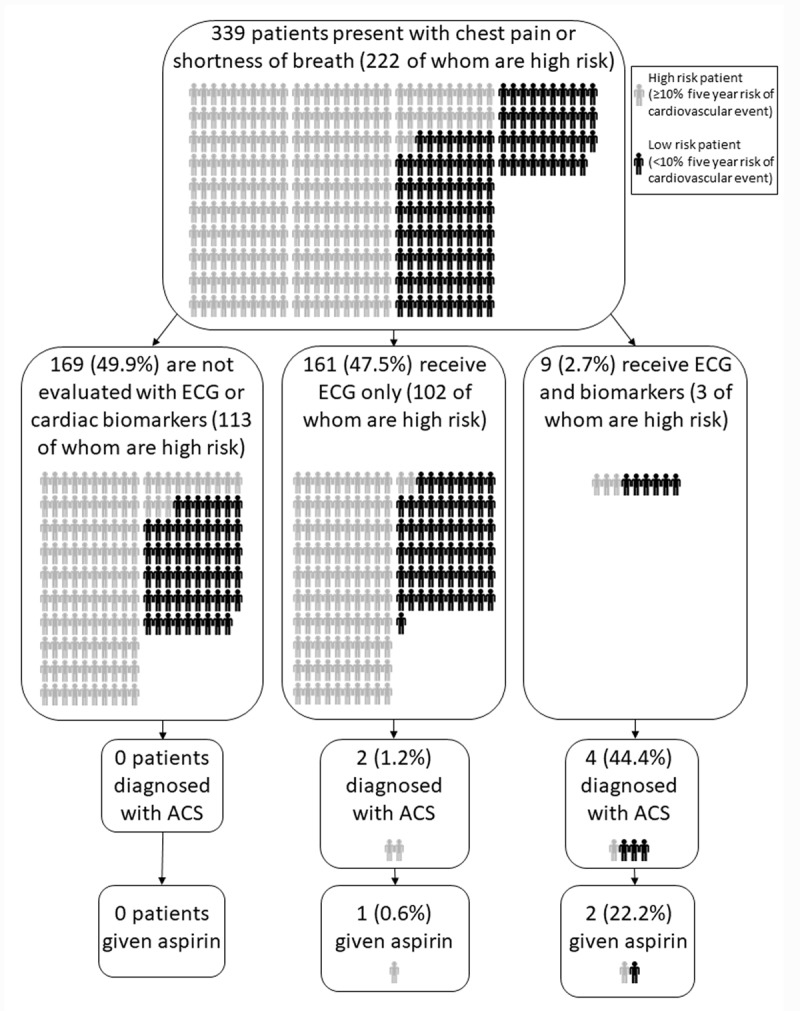
Patterns of diagnosis and treatment for ACS among emergency department patients in northern Tanzania, 2018.

Of participants, 170 (50.1%) underwent ECG testing and 9 (2.7%) underwent cardiac biomarker testing (Table 3). The most common clinical diagnoses were symptomatic hypertension (104 patients, 30.7%) and heart failure (99 patients, 29.2%). Six patients (1.8%) were diagnosed with ACS, and 3 (0.9%) were given aspirin. Approximately one-third of patients (116, 34.2%) were hospitalized. Figure 2 summarizes overall patterns of diagnosis and treatment of ACS among participants.

**Table 3:** Patterns of diagnosis and management of emergency department patients with chest pain or shortness of breath, northern Tanzania, 2018 (N = 339).

	Number of patients	(%)

ECG performed	170	(50.1)
Cardiac biomarkers ordered	9	(2.7)
Other laboratory investigations ordered	246	(72.6)
Treatments administered in the ED		
Aspirin	3	(0.9)
Clopidogrel	2	(0.6)
Furosemide	33	(9.7)
Anti-hypertensive	26	(7.6)
Supplemental oxygen	24	(7.1)
Analgesic	8	(2.4)
Other	23	(6.8)
No medication administered	247	(72.9)
Diagnosis		
Symptomatic hypertension	104	(30.7)
Heart failure	99	(29.2)
PUD/gastritis	27	(8.0)
Non-specific chest pain	14	(4.1)
Pneumonia	12	(3.5)
Asthma/COPD	11	(3.2)
Malignancy	11	(3.2)
Acute coronary syndrome	6	(1.8)
Other	55	(16.2)
Admitted to hospital	116	(34.2)

PUD: peptic ulcer disease. COPD: Chronic obstructive pulmonary disease.

On univariate analysis, patients who received an ECG were more likely to have a primary complaint of chest pain (OR 1.57, 95% CI 1.01–2.47, *p* = 0.046) and less likely to have a history of smoking (OR 0.59, 95% CI 0.37–0.94, *p* = 0.025) than those who did not receive an ECG (Table 4). There was otherwise no association between decision to obtain an ECG and overall five-year cardiovascular risk, age, hypertension, or exertional nature of symptoms. The results of the multivariate logistic regression to identify predictors of decision to obtain ECG are presented in the appendix (Table A.1). On multivariate analysis, the only statistically significant predictor of physician decision to obtain an ECG was lack of smoking history (OR 0.48, 95% CI 0.26–0.88, *p* = 0.018).

**Table 4:** Association between patient characteristics and decision to obtain an ECG for emergency department patients with chest pain or shortness of breath, northern Tanzania, 2018 (N = 339).

	ECG obtained, n (%) (N = 170)	ECG not obtained, n (%) (N = 169)	OR (95% CI)^a^	*p*^b^

Male sex	72 (42.4)	72 (42.6)	0.99 (0.64, 1.52)	0.963
Hypertension	128 (75.3)	124 (73.4)	1.11 (0.68, 1.81)	0.686
Diabetes	18 (10.6)	26 (15.4)	0.65 (0.34, 1.24)	0.189
Hyperlipidemia	24 (14.1)	15 (8.9)	1.68 (0.85, 3.41)	0.130
History of tobacco use	45 (26.5)	64 (37.9)	0.59 (0.37, 0.94)	0.025*
Overweight or obese	84 (49.4)	72 (42.6)	1.31 (0.86, 2.02)	0.209
Personal history of CVD	9 (5.3)	9 (5.3)	0.99 (0.37, 2.64)	0.990
Family history of CVD	46 (27.1)	40 (23.7)	1.20 (0.73, 1.96)	0.473
Poor diet	156 (91.8)	148 (87.6)	1.57 (0.77, 3.29)	0.205
Sedentary lifestyle	65 (38.2)	59 (34.9)	1.15 (0.74, 1.80)	0.525
Primary complaint chest pain	70 (41.2)	52 (30.8)	1.57 (1.01, 2.47)	0.046*
Anginal symptoms	159 (93.5)	155 (91.7)	1.30 (0.57, 3.04)	0.523
Secondary or university education	55 (32.4)	43 (25.4)	1.40 (0.87, 2.25)	0.161
>10% five-year risk of cardiovascular event	109 (64.1)	113 (66.9)	0.89 (0.56, 1.39)	0.595
	**ECG obtained, mean (sd) (N = 170)**	**ECG not obtained, mean (sd) (N = 169)**		***p*^c^**

Age, years	56.9 (18.8)	57.7 (18.7)		0.698
Systolic blood pressure, mmHg	139.2 (29.5)	140.1 (26.6)		0.762
Duration of symptoms, days	30.0 (73.4)	20.6 (31.7)		0.127

^a^ Odds ratios from univariate analyses calculated from 2 × 2 contingency tables. ^b^ Univariate associations between categorical variables assessed via Pearson’s chi-squared. ^c^ Univariate associations between continuous and categorical variables assessed via Welch’s t-test. * *p* < 0.05.

Among admitted patients, the median duration of hospitalization was 7 days (Table 5). Among patients discharged from the ED, the most commonly prescribed medications were antihypertensive medications (84 patients, 37.7%) and furosemide (37 patients, 16.6%). Thirty-day telephone follow-up was obtained for 284 (83.8%) patients. Of these, the majority (211 patients, 74.3%) reported that their symptoms had improved or resolved. Twenty (7.0%) had died within thirty days.

**Table 5:** Outcomes of emergency department patients with chest pain or shortness of breath, northern Tanzania, 2018.

Hospitalized patients (N = 116)	Median	(IQR)

Duration of hospitalization, days	7	(4,8)
**Patients discharged from the ED (N = 223)**	**Number of patients**	**(%)**

Prescriptions given		
Antihypertensive	84	(37.7)
Furosemide	37	(16.6)
Antacid	36	(16.1)
Aspirin	29	(13.0)
Acetaminophen	22	(9.9)
Antimicrobial	22	(9.9)
Clopidogrel	22	(9.9)
NSAID	21	(9.4)
Other prescription	57	(25.6)
**Patients completing telephone follow-up (N = 284)**	**Number of patients**	**(%)**

Clinical status at 30 days		
Symptoms resolved	41	(14.4)
Symptoms improved	170	(59.9)
Symptoms unchanged	47	(16.5)
Symptoms worsened	6	(2.1)
Dead	20	(7.0)

NSAID: Non-steroidal anti-inflammatory drug. ED: Emergency department.

## Discussion

This study is among the first to describe patterns of diagnosis and treatment of ACS in SSA. We found large numbers of patients presenting to the ED with chest pain or shortness of breath, usually several days after symptom onset. Most of these patients were at substantial risk for cardiovascular events, but few of these patients underwent full evaluation for ACS, and ACS testing was not targeted to higher risk patients. Ultimately, very few patients received a diagnosis of ACS, and even fewer were treated with aspirin. These findings demonstrate that at a Tanzanian referral hospital, diagnostic workups for ACS are not routine even among patients with cardiovascular risk factors presenting to the ED. These observations support speculation that physician practices may be contributing to ACS under-detection in SSA.

Chest pain was present in 6.7% of adult ED patients, more than five-fold higher than the prevalence observed elsewhere in Tanzania, and similar to the prevalence observed in EDs in Europe and the United States [5,21,22,23]. The prevalence of chest pain noted in our setting is remarkable, given the recent finding that most Tanzanian adults would not present to a hospital for chest pain [15]. This suggests that the number of patients we observed likely represents only a fraction of those with ACS symptoms in the community.

The median duration of illness prior to hospital presentation was seven days, indicating that even those who seek hospital care for such symptoms do not do so promptly. Such delayed presentation stands in stark contrast to care-seeking behavior in high income countries, where median presentation times for patients with chest pain or other potential ACS symptoms are typically less than four hours [6,28]. Even in other low- and middle-income countries (LMICs) such as Pakistan and India, the majority of patients with possible ACS symptoms presented to hospital within 12 hours [1,20]. To our knowledge, this is the first report of pre-hospital delays among patients with chest pain or shortness of breath in SSA, and the results are concerning. The reasons for the pronounced delay in care-seeking observed in our study are likely myriad, but lack of patient awareness may be contributing to the problem. A recent study found that most Tanzanians did not associate chest pain with cardiovascular diseases like ACS [15,16], suggesting that lack of patient appreciation for the potential seriousness of these symptoms may explain their delayed care-seeking. Further research is needed to understand and intervene upon other barriers to prompt hospital presentation.

By conventional standards, the patients presenting in this study were at high risk for ACS, and would have been evaluated for ACS in high-income settings [8]. A large majority had risk factors like hypertension and approximately two-thirds had an overall five-year risk of cardiovascular event greater than 10%. Despite this, diagnostic workups for ACS were not routine. Only half of enrolled patients underwent ECG testing, and this testing was not targeted to higher risk patients. Fewer than 3% of enrolled patients underwent cardiac biomarker testing, an essential part of the diagnostic workup for ACS. As there have been few other studies of diagnostic workups for chest pain and shortness of breath in SSA, additional research is needed to determine if the care we observed in northern Tanzania is representative of practices across SSA. At a national referral hospital elsewhere in Tanzania, 69% of ED patients with chest pain received an ECG and 39% underwent cardiac biomarker testing [23]. As many hospitals in SSA do not have access to electrocardiograms or cardiac biomarker testing [14], complete diagnostic workups for ACS may be even rarer in other settings.

Consistent with other studies from SSA [2,17], ACS was a rare diagnosis in our study, attributed to only 1.8% of patients with chest pain or shortness of breath. However, given the low rates of ECG and cardiac biomarker testing, it remains unclear whether the scarcity of ACS cases is reflective of low disease burden, frequent misdiagnosis, or both. The majority of patients were diagnosed with symptomatic hypertension or heart failure, two diagnoses that arguably require exclusion of ACS. Although not the focus of our study, the large numbers of ED patients we observed being diagnosed with heart failure or hypertension are consistent with what has been reported elsewhere in Tanzania [9,23], suggesting that these diseases warrant particular attention from public health officials. Less than 1% of enrolled patients received empiric treatment with aspirin, an inexpensive and widely available treatment known to reduce mortality in ACS.

Assessing the appropriateness of the patterns of care observed in our study is difficult without knowledge of the local burden of ACS. Like most countries in SSA, there are currently no data regarding the burden of ACS disease in Tanzania [13,14]. Thus, establishing the prevalence of ACS in this patient population is necessary to develop interventions to improve care. Regardless, our results suggest that physician practices may be contributing to under-detection of ACS in Tanzania. Additional research is needed to understand physician-perceived barriers to ACS diagnosis and care.

Within 30 days of hospital presentation, 7% of enrolled patients had died, nearly 100-fold higher than the 0.08% 30-day mortality rate among the general adult population in Kilimanjaro [25]. This finding suggests urgent interventions are needed to improve diagnosis and care for this patient population, regardless of the underlying cause of their symptoms.

## Limitations

This study had several limitations. First, this study relied on patient self-report to identify risk factors such as diabetes and hyperlipidemia. Given the large proportion of adults in SSA with undiagnosed diabetes and other risk factors [27], this likely resulted in an underestimation of the risk profile of this patient population. Similarly, social desirability bias may have affected participants’ responses to questions regarding cigarette smoking, exercise, and HIV infection, again leading to an underestimation of cardiovascular risk. Additionally, although the kinds of diagnostic investigations ordered by physicians were recorded by the study team, the results of such testing, such as ECG tracings and cardiac biomarker values, were not collected. This data would have led to a better assessment of the appropriateness of the clinical diagnoses and treatments given to enrolled patients, particularly among those diagnosed with ACS. With regards to predictors of decision to obtain an ECG, recent qualitative studies from Tanzania and Kenya found that cost of diagnostic investigations was perceived by providers as an important barrier to ACS care [4,14]. Thus, consideration of patients’ financial capacity may have played a significant role in the decision to obtain an ECG. Unfortunately, individual household income data was not collected during this study, so we are unable to describe the association between personal wealth and decision to obtain an ECG in our study population. Notably, level of education, which may serve as a weak proxy for socioeconomic status, was not significantly associated with decision to obtain an ECG in our study. Furthermore, as this study was conducted in a referral hospital, the patient population and patterns of care observed in our study may not be representative of what would be observed in other kinds of healthcare facilities in Tanzania, where diagnostic equipment for ACS may be unavailable [14]. Finally, we relied on telephone follow-up, which likely resulted in underrepresentation of lower-income patients and patients who had died. This would have yielded an underestimation of the 30-day mortality rate.

In conclusion, large numbers of adults present to the ED in northern Tanzania with chest pain and shortness of breath, but marked delays in care-seeking are common. The majority of these patients are at high risk for cardiovascular disease, but full diagnostic workups for ACS are infrequent and ACS is rarely diagnosed. Further study is needed to understand physician- and patient-related barriers to care and to quantify the true local burden of disease.

## Appendix

**Table A.1:** Results of multivariate logistic regression to identify predictors of decision to obtain an ECG for emergency department patients with chest pain or shortness of breath, northern Tanzania, 2018 (N = 339).

Patient characteristic	ECG obtained, n (%) (N = 170)	ECG not obtained, n (%) (N = 169)	Adjusted OR (95% CI)	*p*

Male sex	72 (42.4)	72 (42.6)	1.40 (0.83, 2.40)	0.211
Hypertension	128 (75.3)	124 (73.4)	1.10 (0.65, 1.87)	0.713
Diabetes	18 (10.6)	26 (15.4)	0.60 (0.30, 1.17)	0.137
History of tobacco use	45 (26.5)	64 (37.9)	0.48 (0.26, 0.88)	0.018*
Primary complaint chest pain	70 (41.2)	52 (30.8)	1.40 (0.88, 2.24)	0.156
	**ECG obtained, mean (sd) (N = 170)**	**ECG not obtained, mean (sd) (N = 169)**	**Adjusted OR (95% CI)**	***p***

Age, years	56.9 (18.8)	57.7 (18.7)	1.01 (0.99, 1.02)	0.361

* *p* < 0.05.

## Abbreviations

ACS: acute coronary syndrome

BMI: body mass index

CCS: Canadian Cardiovascular Society

ECG: electrocardiogram

ED: emergency department

KCMC: Kilimanjaro Christian Medical Centre

SSA: sub-Saharan Africa

WHO: World Health Organization
